# Supplementary material for: Structural insights into insect-selective sodium channel toxins drive AI-enhanced biopesticide design
Source: Nat Commun. 2026 Mar 6;17:3543. doi: 10.1038/s41467-026-70190-z (PMC13087302; doi:10.1038/s41467-026-70190-z)
Supplement: Supplementary file 1 — Supplementary Information [file 41467_2026_70190_MOESM1_ESM.pdf]

# Supplementary Information for

## Structural Insights into Insect-Selective Sodium Channel Toxins Drive AI-Enhanced Biopesticide Design

Heng Jiang[1#], Ruibo Gao[2,3,4#], Huiqin Xu[5#], Cheng Wang[6#], Shuyue Ma[1], Yishu Gong[1], Lianyun Lin[1], Lina Yang[1], Xiang Li[1], Ye Liu[1], Rongcai Lu[2,3,4], Jun-An Ma[7], Jinbo Xu[6], Ke Dong[8], Filip Van Petegem[9], Zheng Liu[5*], Shaoying Wu[2,3,4*], Zhiguang Yuchi[1,10,11*]

[1] State Key Laboratory of Synthetic Biology; Frontiers Science Center for Synthetic Biology; Tianjin Key Laboratory for Modern Drug Delivery & High-Efficiency; School of Pharmaceutical Science and Technology, Faculty of Medicine, Tianjin University, Tianjin, China

[2] School of Breeding and Multiplication (Sanya Institute of Breeding and Multiplication), Hainan University, Sanya, Hainan, China

[3] School of Life and Health Sciences, Hainan University, Haikou, Hainan, China

[4] School of Tropical Agriculture and Forestry (School of Agricultural and Rural Affairs, School of Rural Revitalization), Hainan University, Danzhou, Hainan, China

[5] Cryo-electron Microscopy Center and Department of Pharmacology, School of Medicine, Southern University of Science and Technology, Shenzhen, Guangdong, China

[6] MoleculeMind Inc., Beijing, China

[7] Department of Chemistry, State Key Laboratory Synthetic Biology, Tianjin University, Tianjin, China

[8] Department of Biology, Duke University, Durham, North Carolina, USA

[9] Department of Biochemistry and Molecular Biology, Life Sciences Institute, University of British Columbia, Vancouver, British Columbia, Canada

[10] Haihe Laboratory of Sustainable Chemical Transformations, Tianjin, China

[11] Guangdong Laboratory for Lingnan Modern Agriculture (Shenzhen Branch), Agricultural Genomics Institute at Shenzhen, Chinese Academy of Agricultural Sciences, Shenzhen, Guangdong, China

[#]These authors contributed equally.

[*]Email: liuz3@sustech.edu.cn, wsywsy6000@hainanu.edu.cn, yuchi@tju.edu.cn

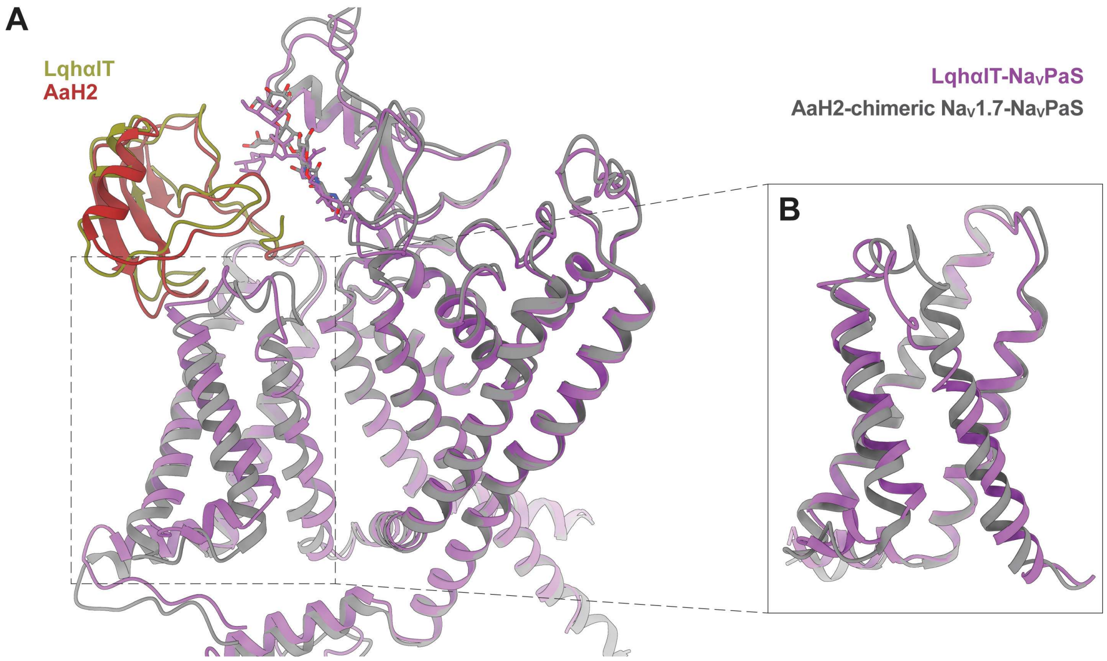


**Supplementary Fig. 1 Comparison of LqhαIT binding site on $Na_vPaS$ and AaH2 binding site on $hNa_v1.7$. (A)** Superposition of $Na_vPaS$-LqhαIT and $hNa_v1.7$-$Na_vPaS$-AaH2 (PDB ID: 6NT4), showing that LqhαIT and AaH2 share a similar binding site. **(B)** The VSD4s of two sodium channels exhibit similar but nonidentical conformation.

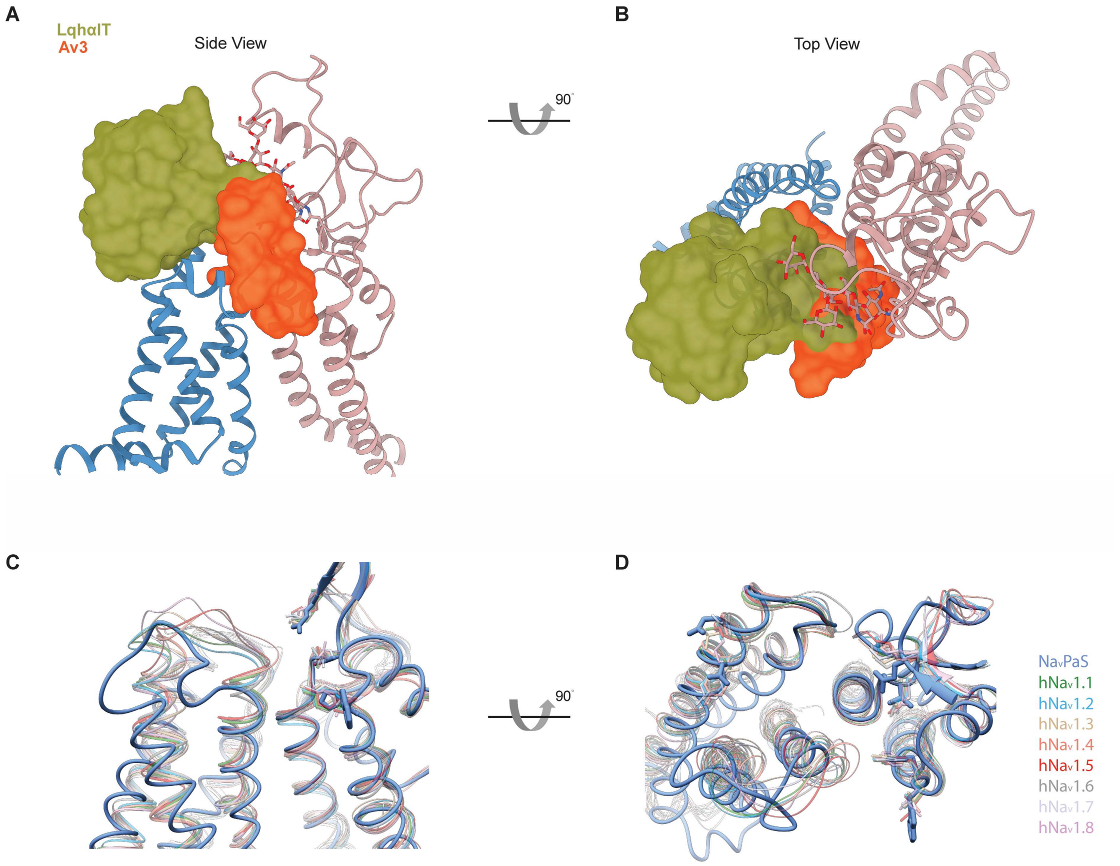


**Supplementary Fig. 2 Structural comparison of Av3 and LqhαIT binding sites. (A, B)** Side (A) and top (B) views of the toxin-binding sites on $Na_vPaS$, showing minimal overlap between the binding interfaces of Av3 and LqhαIT. **(C, D)** Side (C) and top (D) views of the structural superposition of Av3 and LqhαIT binding sites on $Na_vPaS$ with the corresponding regions from human $Na_v$ channels ($hNa_v1.1$-1.8; PDB IDs: 7DTD, 6J8E, 7W77, 6AGF, 7DTC, 8FHD, 7W9K, and 9DBK, respectively), highlighting insect-specific structural features at the toxin-binding interfaces. The high degree of structural conservation among these mammalian isoforms suggests that the observed lack of Av3 and LqhαIT activity on $hNa_v1.5$ likely extends to other human $Na_v$ channels.

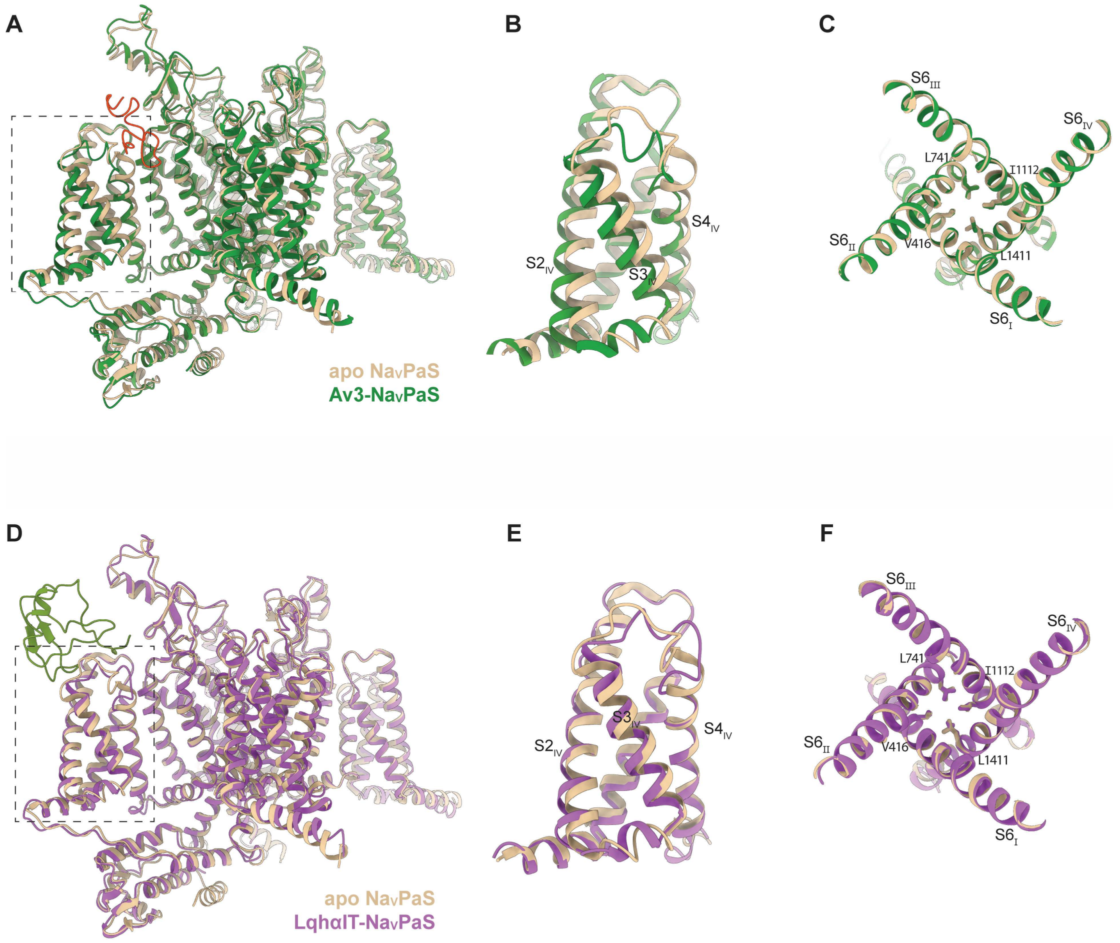


**Supplementary Fig. 3 Pore-based superposition of apo Na$_v$PaS and Na$_v$PaS-Av3 (A-C) and Na$_v$PaS-LqhαIT (D-F), showing conformational changes induced by toxin binding. (A, D)** Side views of the pore-based superposition of Na$_v$PaS in apo state and toxin bound states. **(B, E)** Enlarged view of VSD4, showing the rigid-body displacement for the S2-S4 helices. **(C, F)** Top view of the activation gate, highlighting two layers of hydrophobic residues (shown as sticks) that define the pore constriction, indicating an unaltered pore conformation across the structures.

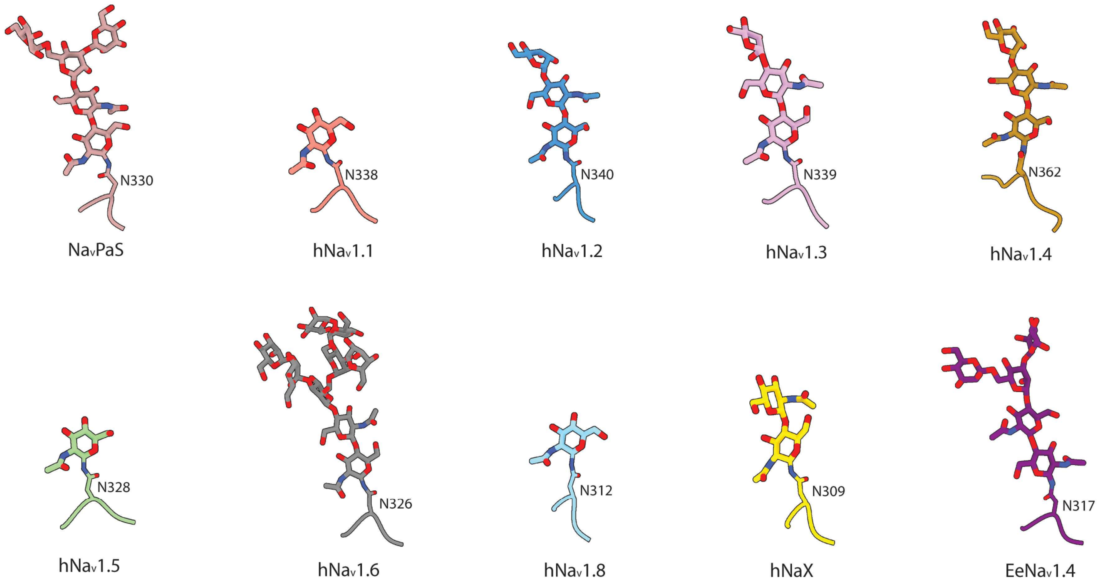


**Supplementary Fig. 4 The toxin-interacting glycan moieties on PD1 from $Na_vPaS$ and other $Na_v$s with determined structures.** Glycan moieties are observed in $hNa_v1.1$ (PDB ID: 7DTD), $hNa_v1.2$ (PDB ID: 6J8E), $hNa_v1.3$ (PDB ID: 7W77), $hNa_v1.4$ (PDB ID: 6AGF), $hNa_v1.5$ (PDB ID: 7DTC), $hNa_v1.6$ (PDB ID: 8FHD), $hNa_v1.8$ (PDB ID: 7WE4), hNaX (PDB ID: 7TJ9), and $EeNa_v1.4$ (PDB ID: 5XSY). Notably, $hNa_v1.7$ lacks this glycosylation site.

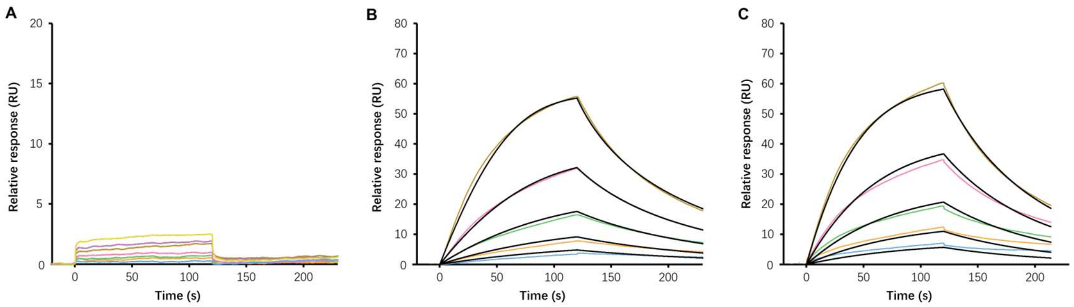


**Supplementary Fig. 5 Representative SPR sensorgrams of LqhαIT binding to $Na_vPaS$. (A)** Sensorgrams showing LqhαIT binding to $Na_vPaS$ in the presence of 0.04% GDN. No detectable binding was observed across the tested concentrations. **(B, C)** Sensorgrams of LqhαIT binding to $Na_vPaS$ collected at the beginning (B) and end (C) of a series of SPR measurements performed on the same immobilized channel while screening multiple mutant constructs. Colored traces represent experimental data, and black lines denote global fits to a 1:1 binding model. The consistent $K_D$ values confirm both the structural stability of the immobilized channel and the reproducibility of the measurements.

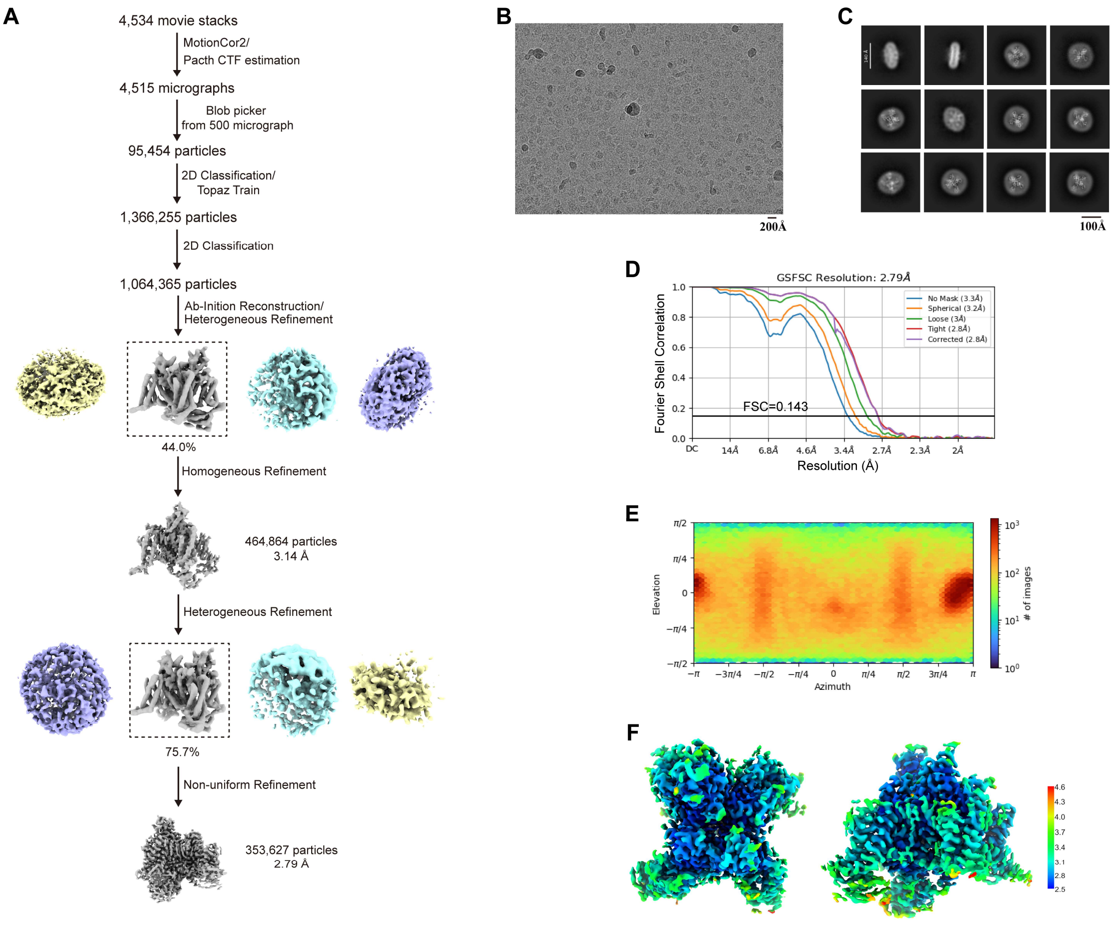


**Supplementary Fig. 6 Cryo-EM data collection, single-particle analysis, and reconstruction of the $Na_V$PaS-Av3 structure. (A)** Flowchart for cryo-EM data processing. **(B)** Representative Cryo-EM micrograph. **(C)** Representative two-dimensional class averages. **(D)** Fourier shell correlation (FSC) curves. **(E)** Orientation distribution plot. **(F)** Local resolution maps.

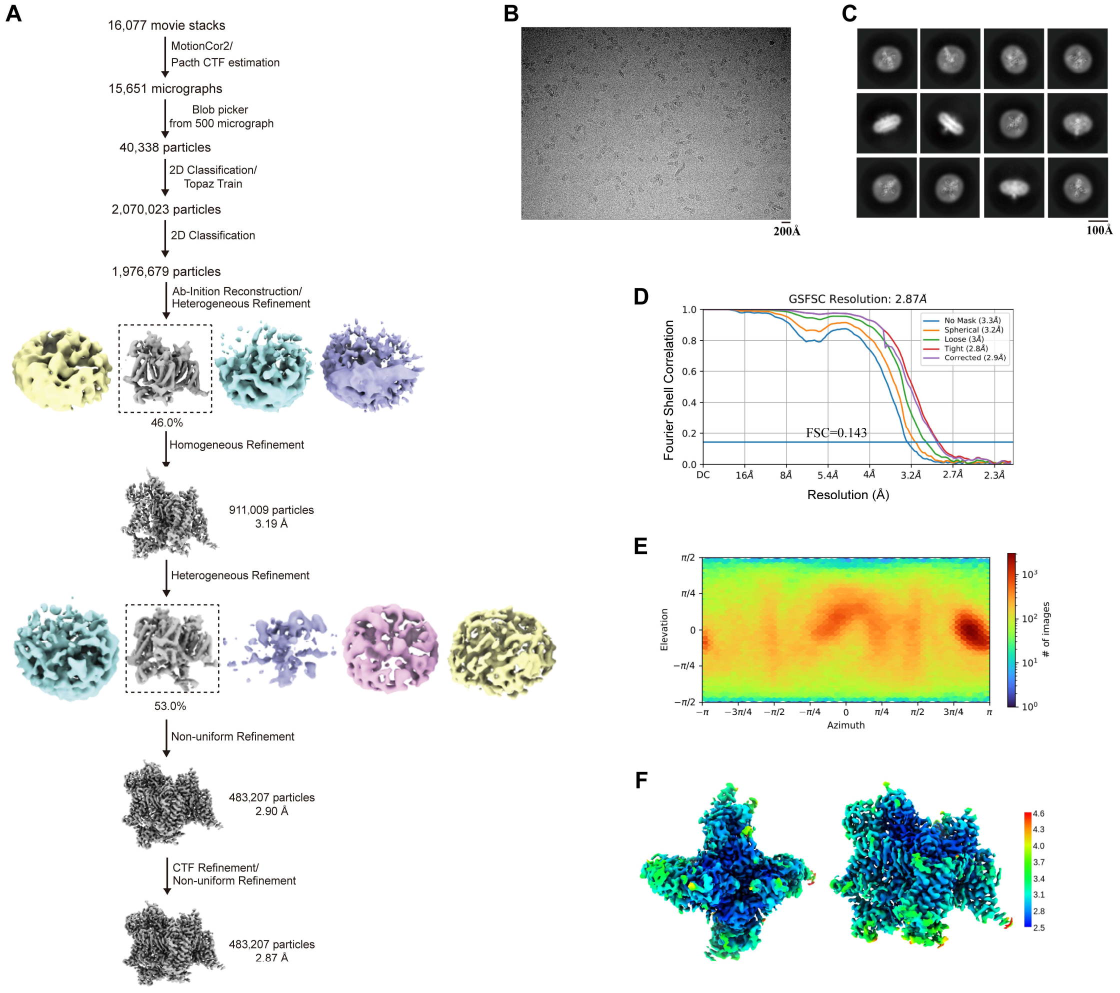


**Supplementary Fig. 7 Cryo-EM data collection, single-particle analysis, and reconstruction of the $Na_vPaS$-LqhαIT structure. (A)** Flowchart for cryo-EM data processing. **(B)** Representative Cryo-EM micrograph. **(C)** Representative two-dimensional class averages. **(D)** FSC curves. **(E)** Orientation distribution plot. **(F)** Local resolution maps.

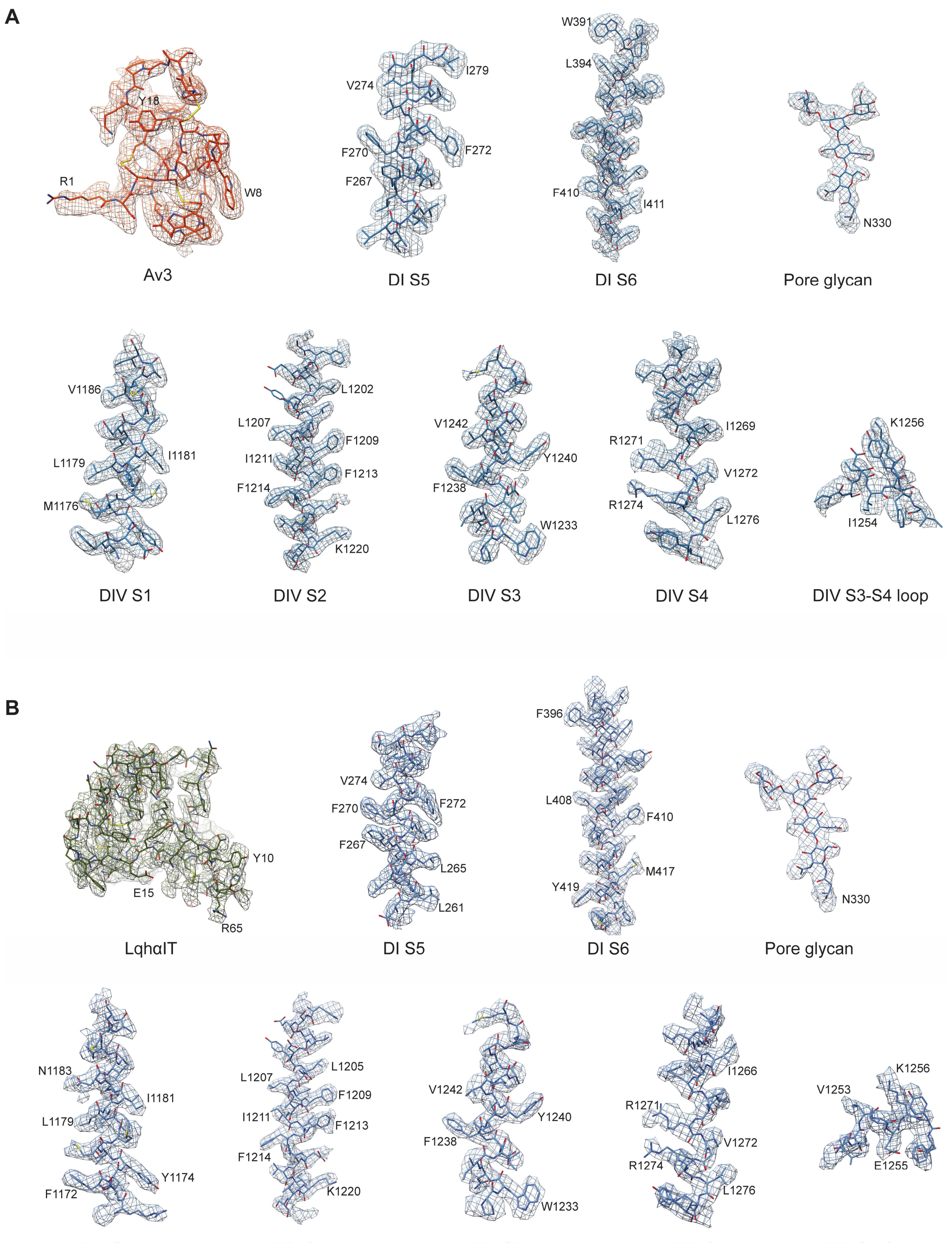


**Supplementary Fig. 8 Representative cryo-EM density maps of $Na_v$PaS-toxin complexes. (A, B)** Cryo-EM density of selected structural elements in the $Na_v$PaS-Av3 (A) and $Na_v$PaS-LqhαIT (B). The maps, contoured at 6σ for visualization, illustrate the density quality for key α-helices and side chains.

**Supplementary Table 1. Kinetic constants and binding affinities of Av3, LqhαIT, and their mutants to $Na_vPaS$, as measured by SPR.** N.D., $K_D$ not detectable.

| | ka (1/Ms) | kd (1/s) | $K_D$ (M) |
|---|---|---|---|
| Av3 | 1.20E+05 | 2.59E-03 | 2.15E-08 |
| P5A | 1.50E+05 | 3.06E-03 | 2.04E-08 |
| Y7A | N.D. | N.D. | N.D. |
| W8A | N.D. | N.D. | N.D. |
| P12A | 7.99E+04 | 4.38E-03 | 5.48E-08 |
| Q15A | 1.04E+05 | 2.12E-03 | 2.04E-08 |
| W13A | N.D. | N.D. | N.D. |
| Y18A | N.D. | N.D. | N.D. |
| LqhαIT | 2.25E+05 | 1.94E-02 | 8.65E-08 |
| K8D | 3.87E+05 | 3.71E-01 | 9.57E-07 |
| N9D | 2.32E+05 | 1.42E-02 | 6.13E-08 |
| Y10V | 1.69E+05 | 5.13E-02 | 3.03E-07 |
| V13T | 1.63E+05 | 2.85E-02 | 1.75E-07 |
| F17G | 2.04E+05 | 8.43E-02 | 4.14E-07 |
| R18D | N.D. | N.D. | N.D. |
| K41P | 2.01E+05 | 5.00E-02 | 2.49E-07 |
| N44A | 3.39E+05 | 1.36E-01 | 4.02E-07 |
| I57T | 4.47E+05 | 1.14E-01 | 2.56E-07 |
| V59G | 3.37E+05 | 3.75E-02 | 1.11E-07 |
| K62R | 2.75E+05 | 2.09E-02 | 7.59E-08 |
| H64A | 2.37E+06 | 2.35E-01 | 9.89E-08 |
| A4W | 1.60E+05 | 4.21E-02 | 2.63E-07 |
| I6Y | 4.65E+04 | 1.64E-02 | 3.53E-07 |
| V13F | N.D. | N.D. | N.D. |
| V13Y | 1.31E+05 | 1.17E-02 | 8.94E-08 |
| E15P | N.D. | N.D. | N.D. |
| E15Q | N.D. | N.D. | N.D. |
| E15F | 3.87E+04 | 7.58E-04 | 1.96E-08 |
| E15S | 2.00E+05 | 1.45E-02 | 7.23E-08 |
| E15I | 1.91E+04 | 2.15E-03 | 1.13E-07 |
| E15M | 3.92E+04 | 3.59E-03 | 9.16E-08 |
| E15Y | 3.15E+07 | 7.70E-01 | 2.44E-08 |
| F17M | N.D. | N.D. | N.D. |
| F17R | N.D. | N.D. | N.D. |
| R18M | 4.87E+04 | 4.50E-02 | 9.25E-07 |
| R18W | 4.34E+04 | 2.35E-02 | 5.41E-07 |
| A20D | 1.93E+05 | 7.34E-02 | 3.81E-07 |

| | ka (1/Ms) | kd (1/s) | $K_D$ (M) |
|---|---|---|---|
| W38G | N.D. | N.D. | N.D. |
| A39L | 3.98E+04 | 1.92E-03 | 4.82E-08 |
| A39H | N.D. | N.D. | N.D. |
| A39S | N.D. | N.D. | N.D. |
| A39N | 7.24E+03 | 3.07E-01 | 4.24E-05 |
| A39W | 1.16E+07 | 6.88E-02 | 5.94E-09 |
| A39Q | N.D. | N.D. | N.D. |
| A39K | N.D. | N.D. | N.D. |
| A39R | 2.88E+04 | 3.42E-03 | 1.19E-07 |
| A50E | N.D. | N.D. | N.D. |
| A50D | 1.26E+05 | 1.84E-02 | 1.46E-07 |
| R65G | 6.46E+05 | 9.91E-03 | 1.53E-08 |

**Supplementary Table 2. Primers for Av3 and LqhαIT mutants.**

| Name | Sequence (5'→ 3') |
|---|---|
| Av3-P5A-F | CGTTCTTGTTGTGCGTGTTATTGGGGTG |
| Av3-P5A-R | CACCCCAATAACACGCACAACAAGAACG |
| Av3-Y7A-F | CTTGTTGTCCATGTGCGTGGGGTGGTTGCC |
| Av3-Y7A-R | GGCAACCACCCCACGCACATGGACAACAAG |
| Av3-W8A-F | GTCCATGTTATGCGGGTGGTTGCCC |
| Av3-W8A-R | GGGCAACCACCCGCATAACATGGAC |
| Av3-P12A-F | TGGGGTGGTTGCGCGTGGGGTCAGAAC |
| Av3-P12A-R | GTTCTGACCCCACGCGCAACCACCCCA |
| Av3-Q15A-F | GCCCATGGGGTGCGAACTGTTACCCAG |
| Av3-Q15A-R | CTGGGTAACAGTTCGCACCCCATGGGC |
| Av3-W13A-F | GTGGTTGCCCAGCGGGTCAGAACTG |
| Av3-W13A-R | CAGTTCTGACCCGCTGGGCAACCAC |
| Av3-Y18A-F | GTCAGAACTGTGCGCCAGAAGGCTG |
| Av3-Y18A-R | CAGCCTTCTGGCGCACAGTTCTGAC |
| LqhαIT-K8D-F | GTGATGCCTATATTGCCGATAATTATAATTGCG |
| LqhαIT-K8D-R | CGCAATTATAATTATCGGCAATATAGGCATCAC |
| LqhαIT-N9D-F | GCCTATATTGCCAAAGATTATAATTGCGTGTACG |
| LqhαIT-N9D-R | CGTACACGCAATTATAATCTTTGGCAATATAGGC |
| LqhαIT-Y10V-F | GCCAAAAATGTGAATTGCGTGTACG |
| LqhαIT-Y10V-R | CGTACACGCAATTCACATTTTTGGC |
| LqhαIT-V13T-F | CAAAAATTATAATTGCACCTACGAATGTTTCCGTG |
| LqhαIT-V13T-R | CACGGAAACATTCGTAGGTGCAATTATAATTTTTG |

| Name | Sequence (5'→ 3') |
|---|---|
| LqhαIT-F17G-F | GCGTGTACGAATGTGGCCGTGATGCATATTG |
| LqhαIT-F17G-R | CAATATGCATCACGGCCACATTCGTACACGC |
| LqhαIT-R18D-F | CGAATGTTTCGATGATGCATATTG |
| LqhαIT-R18D-R | CAATATGCATCATCGAAACATTCG |
| LqhαIT-K41P-F | CAGTGGGCCGGTCCGTATGGTAATGCATG |
| LqhαIT-K41P-R | CATGCATTACCATACGGACCGGCCCACTG |
| LqhαIT-N44A-F | CCGGTAAATATGGTGCGGCATGTTGGTGC |
| LqhαIT-N44A-R | GCACCAACATGCCGCACCATATTTACCGG |
| LqhαIT-I57T-F | GATAATGTGCCGACCCGTGTTCCGGG |
| LqhαIT-I57T-R | CCCGGAACACGGGTCGGCACATTATC |
| LqhαIT-V59G-F | CCGATTCGTGGTCCGGGTAAATG |
| LqhαIT-V59G-R | CATTTACCCGGACCACGAATCGG |
| LqhαIT-K62R-F | CGTGTTCCGGGTGCGTGTCATCGCAAA |
| LqhαIT-K62R-R | TTTGCGATGACACGCACCCGGAACACG |
| LqhαIT-H64A-F | CGGGTAAATGTGCGCGCAAATAAAATATTG |
| LqhαIT-H64A-R | CAATATTTTATTTGCGCGCACATTTACCCG |
| LqhαIT-A4W-F | CCGTGCGTGATTGGTATATTGCCAAAAAT |
| LqhαIT-A4W-R | ATTTTTGGCAATATACCAATCACGCACGG |
| LqhαIT-I6Y-F | CGTGCGTGATGCCTATTATGCCAAAAATTATAATTGC |
| LqhαIT-I6Y-R | GCAATTATAATTTTTGGCATAATAGGCATCACGCACG |
| LqhαIT-V13F-F | GCCAAAAATTATAATTGCTTTTACGAATGTTTCCGTGATGC |
| LqhαIT-V13F-R | GCATCACGGAAACATTCGTAAAAGCAATTATAATTTTTGGC |
| LqhαIT-V13Y-F | GCCAAAAATTATAATTGCTATTACGAATGTTTCCGTGATGC |
| LqhαIT-V13Y-R | GCATCACGGAAACATTCGTAATAGCAATTATAATTTTTGGC |
| LqhαIT-E15P-F | ATAATTGCGTGTACCCGTGTTTCCGTGATG |
| LqhαIT-E15P-R | CATCACGGAAACACGGGTACACGCAATTAT |
| LqhαIT-E15Q-F | ATAATTGCGTGTACCAGTGTTTCCGTGATG |
| LqhαIT-E15Q-R | CATCACGGAAACACTGGTACACGCAATTAT |
| LqhαIT-E15F-F | TTGCGTGTACTTTTGTTTCCGTGATGC |
| LqhαIT-E15F-R | GCATCACGGAAACAAAAGTACACGCAA |
| LqhαIT-E15S-F | ATAATTGCGTGTACAGCTGTTTCCGTGATG |
| LqhαIT-E15S-R | CATCACGGAAACAGCTGTACACGCAATTAT |
| LqhαIT-E15I-F | ATAATTGCGTGTACATTTGTTTCCGTGATG |
| LqhαIT-E15I-R | CATCACGGAAACAAATGTACACGCAATTAT |
| LqhαIT-E15M-F | GCGTGTACATGTGTTTCCGTGATG |
| LqhαIT-E15M-R | CATCACGGAAACACATGTACACGC |
| LqhαIT-E15Y-F | ATAATTGCGTGTACTATTGTTTCCGTGATG |

| Name | Sequence (5'→ 3') |
|---|---|
| LqhαIT-E15Y-R | CATCACGGAAACAATAGTACACGCAATTAT |
| LqhαIT-F17M-F | GTGTACGAATGTATGCGTGATGCATATTG |
| LqhαIT-F17M-R | CAATATGCATCACGCATACATTCGTACAC |
| LqhαIT-F17R-F | GCGTGTACGAATGTCGCCGTGATGCATATTG |
| LqhαIT-F17R-R | CAATATGCATCACGGCGACATTCGTACACGC |
| LqhαIT-R18M-F | CGAATGTTTCATGGATGCATATTG |
| LqhαIT-R18M-R | CAATATGCATCCATGAAACATTCG |
| LqhαIT-R18W-F | CGAATGTTTCTGGGATGCATATTG |
| LqhαIT-R18W-R | CAATATGCATCCCAGAAACATTCG |
| LqhαIT-A20D-F | GTTTCCGTGATGATTATTGTAATG |
| LqhαIT-A20D-R | CATTACAATAATCATCACGGAAAC |
| LqhαIT-W38G-F | GGTTATTGTCAGGGCGCCGGTAAATATG |
| LqhαIT-W38G-R | CATATTTACCGGCGCCCTGACAATAACC |
| LqhαIT-A39L-F | GGTTATTGTCAGTGGCTGGGTAAATATGG |
| LqhαIT-A39L-R | CCATATTTACCCAGCCACTGACAATAACC |
| LqhαIT-A39H-F | GGTTATTGTCAGTGGCATGGTAAATATGG |
| LqhαIT-A39H-R | CCATATTTACCATGCCACTGACAATAACC |
| LqhαIT-A39S-F | GGTTATTGTCAGTGGAGCGGTAAATATGG |
| LqhαIT-A39S-R | CCATATTTACCGCTCCACTGACAATAACC |
| LqhαIT-A39N-F | GGTTATTGTCAGTGGAACGGTAAATATGG |
| LqhαIT-A39N-R | CCATATTTACCGTTCCACTGACAATAACC |
| LqhαIT-A39W-F | GGTTATTGTCAGTGGTGGGGTAAATATGG |
| LqhαIT-A39W-R | CCATATTTACCCCACCACTGACAATAACC |
| LqhαIT-A39Q-F | GGTTATTGTCAGTGGCAGGGTAAATATGG |
| LqhαIT-A39Q-R | CCATATTTACCCTGCCACTGACAATAACC |
| LqhαIT-A39K-F | GGTTATTGTCAGTGGAAAGGTAAATATGG |
| LqhαIT-A39K-R | CCATATTTACCTTTCCACTGACAATAACC |
| LqhαIT-A39R-F | GGTTATTGTCAGTGGCGCGGTAAATATGG |
| LqhαIT-A39R-R | CCATATTTACCGCGCCACTGACAATAACC |
| LqhαIT-A50E-F | GTTGGTGCTATGAACTGCCGGATAATG |
| LqhαIT-A50E-R | CATTATCCGGCAGTTCATAGCACCAAC |
| LqhαIT-A50D-F | GTTGGTGCTATGATCTGCCGGATAATG |
| LqhαIT-A50D-R | CATTATCCGGCAGATCATAGCACCAAC |
| LqhαIT-R65G-F | CGGGTAAATGTCATGGCAAATAAAATATTG |
| LqhαIT-R65G-R | CAATATTTTATTTGCCATGACATTTACCCG |

**Supplementary Table 3. Statistics for data collection and structural refinement of $Na_vPaS$-Av3 and $Na_vPaS$-LqhαIT.**

| | $Na_vPaS$-LqhαIT | $Na_vPaS$-Av3 |
|---|---|---|
| **Data collection and processing** | | |
| Microscope | FEI Titan Krios | FEI Titan Krios |
| Voltage (kV) | 300 | 300 |
| Detector | Gatan K3 Summit | Gatan K3 Summit |
| Magnification | 81,000x | 105,000x |
| Pixel size (Å) | 1.072 | 0.855 |
| Electron exposure ($e^{-}$/Å$^2$) | 50 | 50 |
| Defocus range (μm) | -1.2 to -1.8 | -1.2 to -1.8 |
| Automation software | EPU | EPU |
| Energy filter slit width (eV) | 20 | 20 |
| Micrographs used (no.) | 15,651 | 4,515 |
| Symmetry imposed | C1 | C1 |
| Initial particle images (no.) | 2,070,023 | 1,366,255 |
| Final particle images (no.) | 483,207 | 353,627 |
| Map resolution (Å) | 2.87 | 2.79 |
| FSC threshold | 0.143 | 0.143 |
| Map resolution range (Å) | 2.5 - 4.6 | 2.5 - 3.9 |
| **Refinement** | | |
| Initial model used | 5X0M | 5X0M |
| Model resolution (Å) | 2.8 | 2.7 |
| FSC threshold | 0.143 | 0.143 |
| Map sharpening *B* factor (Å$^2$) | -116.3 | -106.1 |
| Model composition | | |
| Number of atoms | 10,577 | 10,742 |
| Protein residues | 1,310 | 1,349 |
| Ligands | 10 | 11 |
| *B* factors (Å$^2$) | | |
| Protein | 64.62 | 45.58 |
| Ligand | 81.5 | 74.12 |
| R.m.s. deviations | | |
| Bond lengths (Å) | 0.002 | 0.003 |
| Bond angles (°) | 0.524 | 0.668 |
| Validation | | |
| MolProbity score | 1.67 | 1.71 |
| Clash score | 4.51 | 4.07 |
| Rotamer Outliers (%) | 0.09 | 0.09 |
| Ramachandran plot | | |
| Favored (%) | 93.08 | 90.9 |
| Allowed (%) | 6.76 | 8.50 |
| Disallowed (%) | 0.16 | 0.6 |

## Supplementary Note

### Method overview

The overall architecture of ComplexDDG is illustrated in Figure 5B. For a protein-protein complex with a known 3D structure, sequence information is first extracted, and residue-level embeddings are generated for both the wild-type and mutant complexes using the large pretrained protein language model ESM-1v (Evolutionary Scale Modeling)[1]. This process yields feature vectors for each residue that encode information on evolutionary conservation, structural propensity, and functional constraints, providing a rich sequence-level representation for subsequent analysis.

Mutant PDB structures are first modeled using FoldX and refined with PDBFixer v1.8, then parsed together with wild-type structures by Biopython to extract atomic and residue-level structural information[2]. The ComplexDDG framework employs a weight-shared geometric attention network (GAN), in which residue embeddings from ESM-1v are integrated into amino acid node features to generate perturbing-aware representations[3]. This design enables the model to effectively capture synergistic and context-dependent mutation effects, thereby improving the reliability and generalization of predictions across a wide sequence space.

FoldX models the mutant structure using an empirical force field and computes 22 energy terms to quantify physicochemical differences between the wild type and mutant, including total energy, backbone H-bonds, sidechain H-bonds, van der Waals, electrostatics, polar and hydrophobic solvation, *etc.*, generating 22-dimensional energy-difference embeddings. After that, the 22-D energy-difference features are concatenated with the perturbation embeddings derived from the GAN. The combined representation is then passed through three fully connected layers to regress the predicted binding free-energy change ($\Delta\Delta G$).

### Evaluation

We utilized the unified s1131 dataset from the Structural Kinetic and Energetic database of Mutant Protein Interactions (SKEMPI) V2.0 dataset, which consists of 1,131 non-redundant single-point mutations across 144 protein-protein complexes, to evaluate the performance of our model ComplexDDG[4]. Using a fivefold split-by-complex cross-validation strategy, ComplexDDG achieved a Pearson correlation coefficient (PCC) of 0.81 between the predicted and experimental $\Delta\Delta G$ values, demonstrating strong agreement with experimental measurements.

To further assess the performance of ComplexDDG in a comprehensive and fair comparison, we evaluated it alongside five representative models: (1) DDGPredictor[5]: the underlying framework upon which our model is built, used to verify the effectiveness of our improvements; (2) GearBind[6]: a recent model representing the current state-of-the-art performance in this field; (3) Rosetta[7-9]: a widely used protein design suite (version 2024.09+release.06b3cf8ad0), used with default parameters to predict free energy changes; (4) FoldX[2]: a commonly used empirical energy-based protein modeling tool (version 5.0), which makes predictions based on its built-in energy terms; (5) GeoPPI[10]: a highly recognized early geometric model serving as a classical benchmark reference. A split-by-complex fivefold cross-validation strategy was employed to evaluate model performance, using two complementary metrics: (1) Pearson Correlation Coefficient (PCC), measuring the linear correlation between predicted values and experimental values; and (2) Root Mean Square Error (RMSE), quantifying the magnitude of prediction errors.

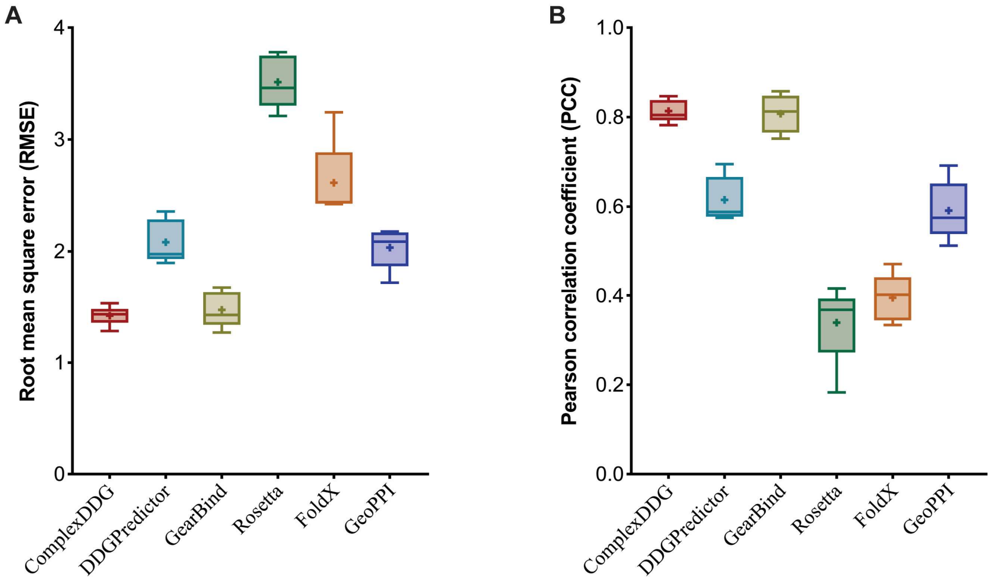


**Supplementary Fig. 9**. **Performance comparison of ComplexDDG versus other methods. (A)** Pearson correlation coefficient (PCC) and **(B)** Root mean square error (RMSE) under fivefold cross-validation on SKEMPI v2.0 s1131. Box plots show the median (center line), interquartile range (box), and 5th-95th percentile range (whiskers); "+" denotes the mean over folds.

As shown in Supplementary Fig. 9 and Supplementary Table 4, ComplexDDG outperformed DDGPredictor, Rosetta, FoldX and GeoPPI in both PCC and RMSE, confirming the effectiveness of the ESM-1v encoding and the FoldX-derived energy-difference features introduced in our model. The performance of ComplexDDG was comparable to GearBind. Compared with GearBind, our model exhibited noticeably narrower interquartile ranges for both metrics, indicating more concentrated performance distributions and reduced variability across multiple tests. These findings

suggest that ComplexDDG achieves near-state-of-the-art performance while providing improved interpretability through multimodal feature integration. Overall, our model demonstrates high competitiveness in predicting binding free-energy change (ΔΔG), making it particularly suitable for applications requiring robust and accurate mutation effect prediction and for downstream experimental design.

**Supplementary Table 4**. **Performance metrics for fivefold cross-validation.** Both PCC and RMSE values are reported for each fold on SKEMPI v2.0 s1131, along with the mean ± SD.

| | Models | Fold 1 | Fold 2 | Fold 3 | Fold 4 | Fold 5 | Mean ± SD |
|---|---|---|---|---|---|---|---|
| **PCC** | ComplexDDG | 0.830 | 0.782 | 0.804 | 0.805 | 0.847 | 0.814 ± 0.025 |
| | DDGPredictor | 0.638 | 0.575 | 0.695 | 0.580 | 0.588 | 0.615 ±0.051 |
| | GearBind | 0.838 | 0.752 | 0.779 | 0.813 | 0.858 | 0.808 ± 0.043 |
| | Rosetta | 0.362 | 0.183 | 0.368 | 0.369 | 0.417 | 0.340 ± 0.090 |
| | FoldX | 0.355 | 0.402 | 0.472 | 0.334 | 0.413 | 0.395 ± 0.054 |
| | GeoPPI | 0.692 | 0.575 | 0.565 | 0.612 | 0.513 | 0.591 ± 0.066 |
| **RMSE** | ComplexDDG | 1.432 | 1.530 | 1.432 | 1.429 | 1.282 | 1.421 ± 0.089 |
| | DDGPredictor | 1.976 | 2.357 | 2.215 | 1.964 | 1.898 | 2.082 ± 0.195 |
| | GearBind | 1.409 | 1.676 | 1.586 | 1.427 | 1.269 | 1.473 ± 0.160 |
| | Rosetta | 3.394 | 3.781 | 3.462 | 3.721 | 3.212 | 3.514 ± 0.236 |
| | FoldX | 2.528 | 2.435 | 2.424 | 3.244 | 2.439 | 2.612 ± 0.354 |
| | GeoPPI | 1.719 | 2.018 | 2.179 | 2.089 | 2.163 | 2.034 ±0.187 |

## Ablation study

To assess the contribution of the two key modules, ESM-1v sequence encoding and FoldX energy-term differences, to model performance, we conducted a systematic ablation analysis. The complete model was used as the baseline, and each module was removed individually while maintaining identical datasets and training conditions.

As shown in Supplementary Fig. 10 and Supplementary Table 5, three configurations were evaluated: (i) w/o ESM-1v: the ESM-1v encoding module was removed, while retaining the FoldX energy-difference and structural features. Under this setting, performance declined (PCC = 0.739, RMSE = 1.712), indicating that the evolutionary and contextual information encoded by ESM-1v is essential for accurate ΔΔG prediction. (ii) w/o FoldX: the ESM-1v encoding was retained but FoldX energy-difference features were excluded. This resulted in a more substantial drop in performance (PCC = 0.630, RMSE = 2.106), demonstrating that the explicit physics-based energy terms provided by FoldX are critical for enhancing predictive accuracy. (iii) Complete Model: integrating both ESM-1v embeddings and FoldX energy-

difference features yielded the best performance (PCC = 0.803, RMSE = 1.476), highlighting their complementary roles in feature representation.

Ablation of the FoldX energy terms caused the largest performance decline (ΔPCC = −0.173, ΔRMSE = +0.630), confirming that physics-based energy priors remain indispensable for capturing accurate binding free-energy changes. Removing the ESM-1v encoding also led to a noticeable reduction in performance (ΔPCC = −0.064, ΔRMSE = +0.236), emphasizing its importance in modeling the evolutionary and contextual landscape of protein sequences. Together, these results demonstrate that integrating explicit physical energy terms with implicit sequence-based evolutionary representations effectively combines the strengths of physics-informed and data-driven learning, thereby significantly improving the accuracy, robustness, and generalization of ΔΔG predictions.

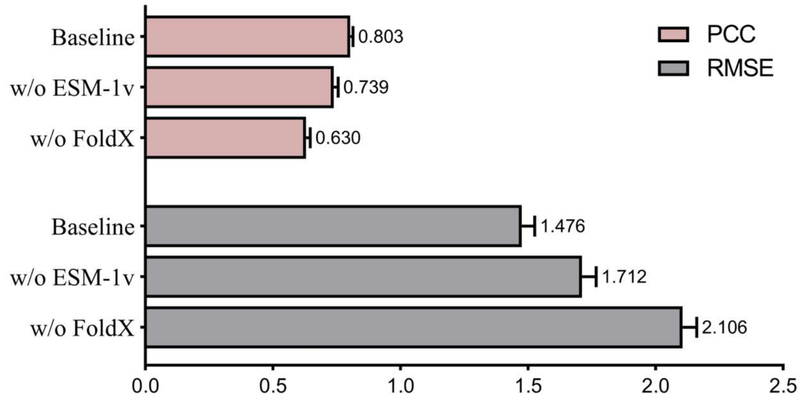


**Supplementary Fig. 10. Ablation study of ComplexDDG.** Bars show mean ± SD across n = 3 independent runs under identical data splits and preprocessing. w/o stands for without.

**Supplementary Table 5. Per-run ablation results of ComplexDDG.** Each variant reports PCC and RMSE across three independent runs. Values in this table are the numeric source for Supplementary Fig. 10.

| **Model variant** | **PCC** | | | **RMSE** | | |
|---|---|---|---|---|---|---|
| | Run 1 | Run 2 | Run 3 | Run 1 | Run 2 | Run 3 |
| Baseline | 0.814 | 0.793 | 0.803 | 1.421 | 1.521 | 1.486 |
| w/o FoldX energy terms | 0.646 | 0.614 | 0.631 | 2.058 | 2.166 | 2.094 |
| w/o ESM-1v embeddings | 0.752 | 0.720 | 0.746 | 1.665 | 1.772 | 1.700 |

## Supplementary References


1. Meier J, Rao R, Verkuil R, Liu J, Sercu T, Rives A. Language models enable zero-shot prediction of the effects of mutations on protein function. *Adv Neur In* **34**, (2021).

2. Delgado J, Radusky LG, Cianferoni D, Serrano L. FoldX 5.0: working with RNA, small molecules and a new graphical interface. *Bioinformatics* **35**, 4168-4169 (2019).

3. Wu F, Wu L, Radev D, Xu J, Li SZ. Integration of pre-trained protein language models into geometric deep learning networks. *Commun Biol* **6**, 876 (2023).

4. Jankauskaite J, Jimenez-Garcia B, Dapkunas J, Fernandez-Recio J, Moal IH. SKEMPI 2.0: an updated benchmark of changes in protein-protein binding energy, kinetics and thermodynamics upon mutation. *Bioinformatics* **35**, 462-469 (2019).

5. Shan S*, et al.* Deep learning guided optimization of human antibody against SARS-CoV-2 variants with broad neutralization. *Proc Natl Acad Sci U S A* **119**, e2122954119 (2022).

6. Cai H*, et al.* Pretrainable geometric graph neural network for antibody affinity maturation. *Nat Commun* **15**, 7785 (2024).

7. Maguire JB*, et al.* Perturbing the energy landscape for improved packing during computational protein design. *Proteins* **89**, 436-449 (2021).

8. Khatib F*, et al.* Algorithm discovery by protein folding game players. *Proc Natl Acad Sci U S A* **108**, 18949-18953 (2011).

9. Tyka MD*, et al.* Alternate states of proteins revealed by detailed energy landscape mapping. *J Mol Biol* **405**, 607-618 (2011).

10. Liu X, Luo Y, Li P, Song S, Peng J. Deep geometric representations for modeling effects of mutations on protein-protein binding affinity. *PLoS Comput Biol* **17**, e1009284 (2021).
