# Supplementary material for: Structural insights into insect-selective sodium channel toxins drive AI-enhanced biopesticide design
Source: Nat Commun. 2026 Mar 6;17:3543. doi: 10.1038/s41467-026-70190-z (PMC13087302; doi:10.1038/s41467-026-70190-z)
Supplement: Supplementary file 2 — Description of Additional Supplementary Files [file 41467_2026_70190_MOESM2_ESM.pdf]

**Description of Additional Supplementary Files**

File name: Supplementary Movie 1
Description: Conformational changes of $Na_v$PaS upon Av3/LqhαIT binding.
