# Supplementary material for: Structural insights into insect-selective sodium channel toxins drive AI-enhanced biopesticide design
Source: Nat Commun. 2026 Mar 6;17:3543. doi: 10.1038/s41467-026-70190-z (PMC13087302; doi:10.1038/s41467-026-70190-z)
Supplement: Supplementary file 4 — Reporting Summary [file 41467_2026_70190_MOESM4_ESM.pdf]

nature portfolio

Corresponding author(s): Zhiguang Yuchi

Last updated by author(s): Feb 18, 2026

Reporting Summary

Nature Portfolio wishes to improve the reproducibility of the work that we publish. This form provides structure for consistency and transparency in reporting. For further information on Nature Portfolio policies, see our Editorial Policies and the Editorial Policy Checklist.

Statistics

For all statistical analyses, confirm that the following items are present in the figure legend, table legend, main text, or Methods section.

n/a	Confirmed	
☐	☒	The exact sample size (n) for each experimental group/condition, given as a discrete number and unit of measurement
☐	☒	A statement on whether measurements were taken from distinct samples or whether the same sample was measured repeatedly
☐	☒	The statistical test(s) used AND whether they are one- or two-sided<br>*Only common tests should be described solely by name; describe more complex techniques in the Methods section.*
☒	☐	A description of all covariates tested
☒	☐	A description of any assumptions or corrections, such as tests of normality and adjustment for multiple comparisons
☐	☒	A full description of the statistical parameters including central tendency (e.g. means) or other basic estimates (e.g. regression coefficient) AND variation (e.g. standard deviation) or associated estimates of uncertainty (e.g. confidence intervals)
☐	☒	For null hypothesis testing, the test statistic (e.g. F, t, r) with confidence intervals, effect sizes, degrees of freedom and P value noted<br>*Give P values as exact values whenever suitable.*
☒	☐	For Bayesian analysis, information on the choice of priors and Markov chain Monte Carlo settings
☐	☒	For hierarchical and complex designs, identification of the appropriate level for tests and full reporting of outcomes
☐	☒	Estimates of effect sizes (e.g. Cohen's d, Pearson's r), indicating how they were calculated

Our web collection on statistics for biologists contains articles on many of the points above.

Software and code

Policy information about availability of computer code

Data collection	Thermo Scientific EPU software, Biacore Insight Evaluation 4.0.8.19879, pClamp 10.6, ESM2, A3D2.0, ComplexDDG
Data analysis	Cryosparc 4.4.1, Coot-0.8.9.2, PHENIX-1.16, UCSF Chimera 1.14, Chimera X1.9, Graphpad Prism 8

For manuscripts utilizing custom algorithms or software that are central to the research but not yet described in published literature, software must be made available to editors and reviewers. We strongly encourage code deposition in a community repository (e.g. GitHub). See the Nature Portfolio guidelines for submitting code & software for further information.

Data

Policy information about availability of data

All manuscripts must include a data availability statement. This statement should provide the following information, where applicable:

- Accession codes, unique identifiers, or web links for publicly available datasets
- A description of any restrictions on data availability
- For clinical datasets or third party data, please ensure that the statement adheres to our policy

The cryo-EM maps have been deposited in the Electron Microscopy Data Bank (EMDB) under accession codes EMD-63108 [https://www.ebi.ac.uk/pdbe/entry/emdb/EMD-63108] (NavPaS-Av3); and EMD-63189 [https://www.ebi.ac.uk/pdbe/entry/emdb/EMD-63189] (NavPaS-LqhαIT). The atomic coordinates have been deposited in the Protein Data Bank (PDB) under accession codes 9LHZ [https://doi.org/10.2210/pdb9LHZ/pdb] (NavPaS-Av3); and 9LKZ [https://doi.org/10.2210/pdb9LKZ/pdb] (NavPaS-LqhαIT). The structures used in this paper are available in the PDB database under accession codes 1ANS [https://doi.org/10.2210/pdb1ANS/

pdb], 2ASC [https://doi.org/10.2210/pdb2ASC/pdb], 5X0M [https://doi.org/10.2210/pdb5X0M/pdb], 6NT4 [https://doi.org/10.2210/pdb6NT4/pdb], 7DTD [https://doi.org/10.2210/pdb7DTD/pdb], 6J8E [https://doi.org/10.2210/pdb6J8E/pdb], 7W77 [https://doi.org/10.2210/pdb7W77/pdb], 6AGF [https://doi.org/10.2210/pdb6AGF/pdb], 7DTC [https://doi.org/10.2210/pdb7DTC/pdb], 8FHD [https://doi.org/10.2210/pdb8FHD/pdb], 7WE4 [https://doi.org/10.2210/pdb7WE4/pdb], 7TJ9 [https://doi.org/10.2210/pdb7TJ9/pdb], and 5XSY [https://doi.org/10.2210/pdb5XSY/pdb]. Source data are provided with this paper.

Research involving human participants, their data, or biological material

Policy information about studies with human participants or human data. See also policy information about sex, gender (identity/presentation), and sexual orientation and race, ethnicity and racism.

Reporting on sex and gender	This information has not been collected.
Reporting on race, ethnicity, or other socially relevant groupings	This information has not been collected.
Population characteristics	This information has not been collected.
Recruitment	This information has not been collected.
Ethics oversight	This information has not been collected.

Note that full information on the approval of the study protocol must also be provided in the manuscript.

Field-specific reporting

Please select the one below that is the best fit for your research. If you are not sure, read the appropriate sections before making your selection.

☒ Life sciences ☐ Behavioural & social sciences ☐ Ecological, evolutionary & environmental sciences

For a reference copy of the document with all sections, see nature.com/documents/nr-reporting-summary-flat.pdf

Life sciences study design

All studies must disclose on these points even when the disclosure is negative.

Sample size	Sample sizes were chosen based on established protocols and prior literature in the field to ensure reproducibility and scientific validity. For cryo-EM experiments, the number of micrographs collected was determined by prior experience and cryo-EM instrument time allocation, ensuring sufficient particle numbers for high-resolution reconstruction. SPR assays were performed using at least five concentrations per toxin variant to evaluate interactions. Electrophysiological recordings were performed in triplicate using three independent cells per condition. For insect bioassays, 24 larvae per toxin concentration were used, which is sufficient to assess toxicity and is consistent with standard practice in insect bioassays.
Data exclusions	Initial cryo-EM images are screened manually to exclude those with low contrast, thick ice or severe ice contaminations, which is a standard procedure for cryo-EM data processing.
Replication	All attempts at replication were successful. Each key finding was supported by at least three independent replicates with similar results. Each cryo-EM dataset includes thousands of particles, providing inherent replication. SPR assays were performed once with ≥5 concentrations per condition, serving as a preliminary high-throughput screen for follow-up validation. These data were not used for statistical comparison, and no replicates were performed. Electrophysiological experiments and insect bioassays were independently repeated three times and successfully reproduced.
Randomization	Allocation of Samples/Organisms/Participants into experimental groups was not performed in this study, therefore randomization is not relevant to our study.
Blinding	The investigators were blinded to group allocation during both data collection and analysis. To ensure impartiality, all procedures and data processing were performed using objective, standardized protocols applied identically across all experimental groups.

Reporting for specific materials, systems and methods

We require information from authors about some types of materials, experimental systems and methods used in many studies. Here, indicate whether each material, system or method listed is relevant to your study. If you are not sure if a list item applies to your research, read the appropriate section before selecting a response.

Materials & experimental systems

n/a	Involved in the study
☒	☐ Antibodies
☐	☒ Eukaryotic cell lines
☒	☐ Palaeontology and archaeology
☐	☒ Animals and other organisms
☒	☐ Clinical data
☒	☐ Dual use research of concern
☒	☐ Plants

Methods

n/a	Involved in the study
☒	☐ ChIP-seq
☒	☐ Flow cytometry
☒	☐ MRI-based neuroimaging

Eukaryotic cell lines

Policy information about cell lines and Sex and Gender in Research

Cell line source(s)	Human Embryonic Kidney 293T cells (ATCC)
Authentication	None of the cell lines have been authenticated.
Mycoplasma contamination	Cell lines were not tested for mycoplasma contamination but no indication of contamination was observed.
Commonly misidentified lines (See ICLAC register)	No commonly misidentified cell lines were used.

Animals and other research organisms

Policy information about studies involving animals; ARRIVE guidelines recommended for reporting animal research, and Sex and Gender in Research

Laboratory animals	Xenopus laevis and Last-instar Galleria mellonella larvae were used in this study.
Wild animals	No wild animals were used in this study.
Reporting on sex	Larval sex was not identified as it is not readily distinguishable at this developmental stage and was not a variable in this study.
Field-collected samples	No field-collected samples were used in this study.
Ethics oversight	All procedures involving Xenopus oocytes were approved by Hainan University Institutional Animal Use and Care Committee and conducted in accordance with institutional guidelines and applicable national regulations of China.

Note that full information on the approval of the study protocol must also be provided in the manuscript.

Plants

Seed stocks	No involvement of plant-related elements in this study.
Novel plant genotypes	No involvement of plant-related elements in this study.
Authentication	No involvement of plant-related elements in this study.
